# Intelligent Fish Recognition Method Based on Variable-Step Size Learning Rate Optimization Strategy

**DOI:** 10.3390/foods14183274

**Published:** 2025-09-21

**Authors:** Yang Liu, Haixu Sui, Feng Liu, Xu Zhang, Xiaoyu Xu, Huihui Wang

**Affiliations:** School of Mechanical Engineering, Dalian Polytechnic University, Dalian 116034, China; 15830829396@163.com (H.S.); 15516644537@163.com (F.L.); zhangxu_dlut@163.com (X.Z.); xuxiaoyu@dlpu.edu.cn (X.X.)

**Keywords:** classification of fish, convolutional neural network, parameter optimization, learning rate

## Abstract

Fish capture usually requires classification of fish species, and the cost of manual classification is relatively high. Recently, deep learning has been widely applied in the fishery field. Transfer learning was conducted on ResNet18, ShuffleNet, EfficientNet, MobileNetV3, and YOLOv8. Through analysis of the influence of the law of learning rate on accuracy during the network learning process, a variable-step learning rate optimization strategy was proposed. Experimental results indicate that the optimal learning rates for fish classification utilizing this strategy were determined to be 0.01, 0.015, 0.001, 0.001, and 0.006 for ResNet18, ShuffleNet, EfficientNet, MobileNetV3, and YOLOv8, respectively. The recognition accuracy rates on the sample set reach 96.33%, 96.74%, 97.50%, 86.73%, 88.49%, respectively, and the average recognition accuracy rate between the sample set and other multi-species interfering fish reaches 93.13%, 93.44%, 96.13%, 95.21%, and 92.16%, respectively. This enables high-precision and rapid sorting of the target fish and other multi-species interfering fish. Compared with global optimization, the number of optimizations can be reduced by more than 97.1%; and compared with the same number of optimizations, the accuracy can be improved by more than 34.21%, which improves the efficiency and accuracy of network training and provides a theoretical reference for the setting of learning rate during model training in the field of deep learning.

## 1. Introduction

In fishery production, the identification and classification of multiple fish species are essential. Manual sorting can significantly impact fishing efficiency and economic benefits. With the large-scale development of offshore fishing operations, traditional manual sorting methods can no longer meet the demands of efficient and precise fishery production. In mechanized fishing operations, catches are typically processed preliminarily on board the ships, making lightweight real-time automated sorting a critical component for enhancing operational efficiency. Therefore, establishing an intelligent fish classification system can not only reduce labor requirements onboard but also improve fishing operation efficiency. This study focuses on common marine-caught fish species as research subjects, with particular emphasis on intelligent recognition methods for mixed fish species.

Numerous scholars have conducted extensive research on fish sorting based on traditional feature extraction methods [1,2,3]. They classified fish by extracting features such as contour, texture, and color. For instance, Wu Jianhui et al. established a mathematical model for the fish body’s back contour by calculating its outline information. Through the interval distribution of the bending potential value of different species’ fish backs, they achieved automatic fish recognition with an accuracy rate of 96% through experimental verification [1]. Yao Runlu et al. extracted texture and morphological features from fish back and fish maw images, which were then fed into a BP neural network for recognition [2]. The final recognition rate reached 75%.

In recent years, the use of convolutional neural networks (CNN) for fish sorting and identification has gained significant attention from scholars [4,5,6,7,8,9,10,11,12,13,14,15]. Wang Wencheng et al. proposed a fish image recognition algorithm based on ResNet50, achieving a classification and recognition accuracy of 93.33% for 10 kinds of marine fish [4]. Vaneeda Allken was able to classify blue whitefish, Atlantic herring and Atlantic mackerel with 94 percent accuracy using deep learning [5]. Lin Meng developed an underwater drone with a 360-degree panoramic camera that serves as the drone’s “eyes,” and through deep learning, fish recognition accuracy reached 87 percent [6]. Ashkan Banan has developed an intelligent, real-time, lossless deep learning neural network approach that achieves the highest accuracy of 100% after 5× cross-verification [12].

In summary, traditional feature extraction methods predominantly rely on manually designed features and impose stringent requirements on image preprocessing (e.g., background treatment), making them inadequate for addressing challenges such as feature transfer and poor generalization. In contrast, deep learning-based fish recognition and sorting methods can autonomously learn feature representations and establish intrinsic correlations through network iterations, with the trained models demonstrating strong generalization capabilities [16].

However, current deep learning architectures generally demand substantial hardware computational power. Given the constraints of offshore operations, the detection equipment installed onboard is typically compact, highly reliable, and low-performance, with limited computational capacity and data processing capabilities that cannot support the intensive computations required by large-scale network models. Furthermore, current parameter optimization during training predominantly employs empirical or adaptive approaches, which are prone to converge to local optima. Consequently, there remains significant room for improvement in model training methodologies.

Therefore, this paper takes perch, cod and snapper, which are common economic fish in Bohai Sea and Yellow Sea, as the research objects. In order to optimize the learning rate in the training process, two lightweight networks were selected for transfer learning based on dataset image preprocessing. Simultaneously, the verification set accuracy under different learning rate conditions was analyzed, and the optimization method for global learning rate parameters was investigated.

## 2. Materials and Methods

In order to achieve accurate identification and classification of snapper, cod and perch, datasets for the above fish species were collected in this study. The acquired image data is carefully screened and preprocessed to ensure its quality. A suitable network model was then selected for the identification and classification of the three fish species.

**Figure 1:**
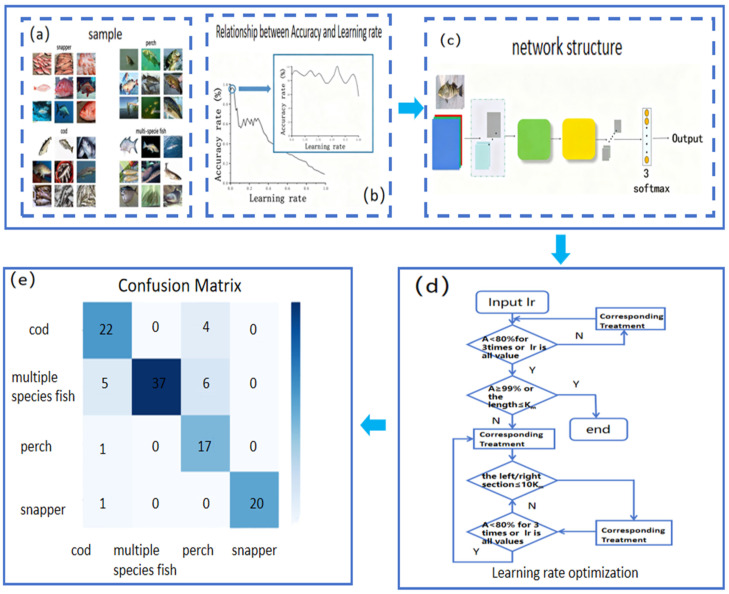
The main flow of the research. (**a**) Fish species dataset. (**b**) Relationship between learning rate and accuracy. (**c**) Simplified diagram of model architecture. (**d**) Learning rate optimization flowchart. (**e**) Confusion Matrix.

The overall technical roadmap of the paper is shown in Figure 1.

### 2.1. Data Source

**Figure 2:**
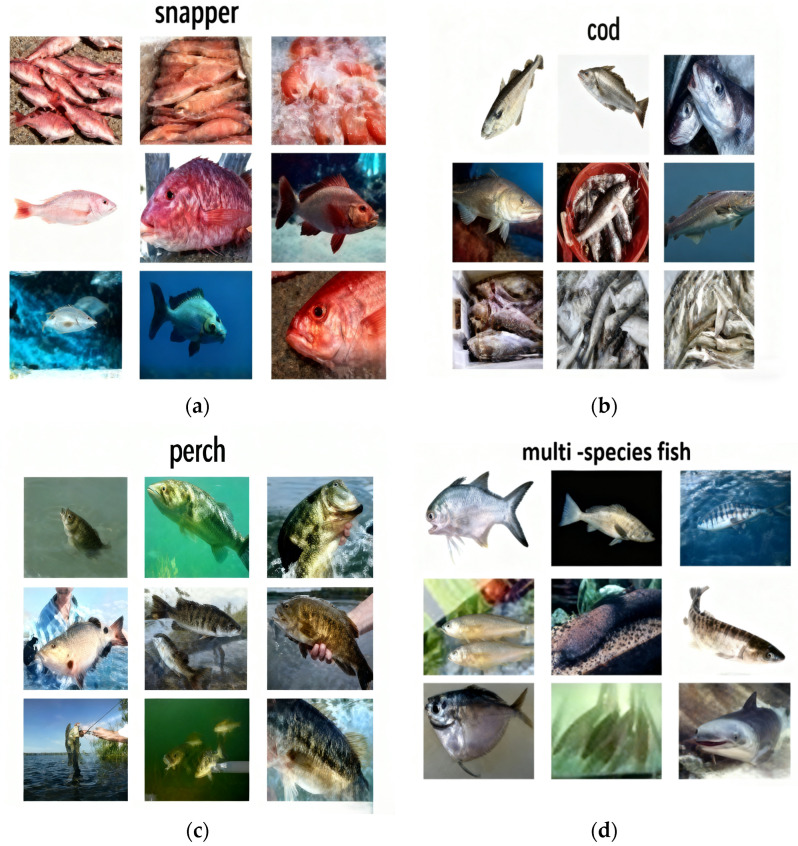
Sample fish picture. (**a**) Snapper; (**b**) cod; (**c**) perch; (**d**) multi-species fish.

To ensure the diversity of fish sample data, this study constructed a comprehensive fish dataset in a complex background environment, as depicted in Figure 2. The dataset comprises images collected from various sources, including contest websites, web crawlers, and a self-built automatic image acquisition system.

All datasets used are open-source and pose no copyright issues.

### 2.2. Data Partitioning

The composition of the image dataset is presented in Table 1. The dataset includes a total of 2289 images, which are divided into training, verification, and test sets. The test set consists of 320 photos, with each category (other multi-species interfering fish, snapper, cod, and perch) having 80 photos. The remaining 1969 images were divided into a 75% training set and a 25% verification set.

### 2.3. Data Preprocessing

To enhance data diversity and reliability while improving the model’s generalization capability, certain data augmentation techniques were employed [17]. Firstly, random flipping was performed along the *X*-axis and *Y*-axis of the images. Secondly, the images were randomly rotated within the range of −90° to 90°.

To ensure standardized and detectable features in the image data, this study preprocessed the original images by resizing them into RGB three-channel images with dimensions of 224 × 224 pixels. The images were then normalized and mapped to the interval [0, 1]. x is the original input value (the data point to be normalized); min is the minimum value in the original dataset; max is the maximum value in the original dataset; $X^{*}$ is the normalized output value (within the range [0, 1]). The mapping function used is as follows:

(1) $$X^{*}=\frac{x-min}{max-min}$$

### 2.4. Lightweight Neural Networks

Given the urgent need for target classification and identification in agricultural production and the limitations of current hardware systems in effectively supporting complex recognition algorithms, this study employs ResNet18, ShuffleNet, EfficientNet, MobileNetV3, and YOLOv8 architectures as lightweight network models for transfer learning.

**Figure 3:**
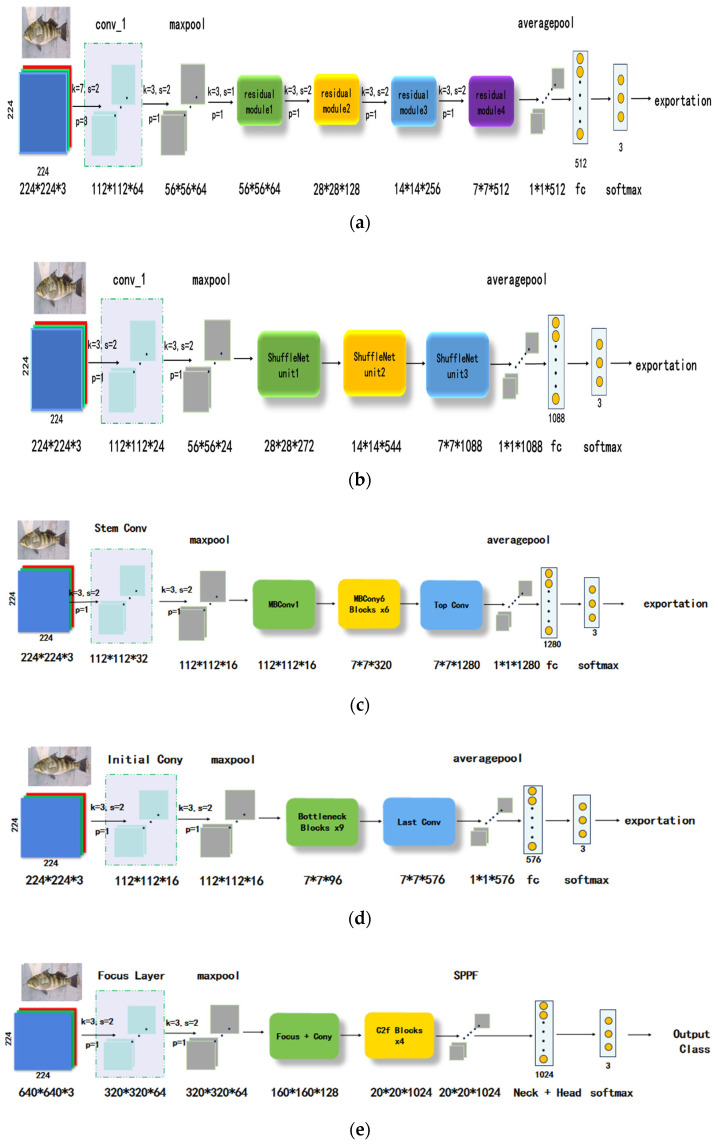
Structure of network. (**a**) Model structure of ResNet18; (**b**) model structure of ShuffleNet; (**c**) model structure of EfficientNet; (**d**) model structure of MobileNetV3; (**e**) model structure of YOLOv8.

ResNet18 utilizes a global 3 × 3 convolution kernel, and its core is the residual module. By leveraging shortcut connections, ResNet18 establishes an identity mapping from input to output information. This not only improves the efficiency of information flow but also mitigates issues such as vanishing gradients and degradation associated with deep networks. The main concept behind ShuffleNet is pointwise group convolution and channel shuffle. Group convolution effectively reduces network capacity, making it more lightweight. Channel rearrangement addresses the accuracy degradation caused by boundary effects resulting from multiple group convolutions. The core idea of EfficientNet is compound model scaling. While traditional methods often arbitrarily scale network depth, width, or input image resolution, EfficientNet uniformly scales these three dimensions using a set of fixed scaling coefficients, achieving an optimal balance between model accuracy and efficiency under limited computational resources. MobileNetV3 combines the advantages of network architecture search and human design, aiming to further optimize the trade-off between accuracy and latency. Its architecture is based on inverted residual structures and linear bottleneck layers, and it introduces a lightweight attention mechanism—an optimized version of the Squeeze-and-Excitation module—as well as the h-swish activation function to enhance performance. YOLOv8 is not a traditional classification network but an advanced all-in-one object detection architecture. Its core lies in an anchor-free detection mechanism and an efficient label assignment strategy, which simplify the training process and improve detection accuracy. The network structure can be visualized in Figure 3.

### 2.5. Transfer Learning

For a three-class classification problem, the fully connected and classified layers of ResNet-18, ShuffleNet, EfficientNet, MobileNetV3, and YOLOv8 networks were all replaced to ensure compatibility between the output dimension and the number of classes. The original output dimension of the fully connected layers in all lightweight neural networks was set to 3 to match the required number of categories. The weight parameters of the aforementioned networks were correspondingly retrained.

To prevent overfitting, the Stochastic Gradient Descent with Momentum (SGDM) optimization algorithm was selected, and a loss function with L2 regularization was adopted. This strategy aims to enhance the generalization capability of the networks and achieve a convergent, stable, and robust objective function.

During the network training process, hyperparameter settings—such as learning rate, batch size, and the number of training epochs—directly affect both the training cycle and model accuracy. Among these, the learning rate has the most significant impact on training efficiency and final classification performance. To achieve higher classification accuracy, the number of training epochs was empirically set to 20 based on verification set performance, and the weight decay coefficient for L2 regularization was correspondingly configured.

Furthermore, the learning rate parameter was systematically investigated to determine the value that yields the highest accuracy with the fewest optimization iterations across the EfficientNet, MobileNetV3, and YOLOv8 models.

### 2.6. Variable-Step Size Learning Rate Optimization Strategy

**Figure 4:**
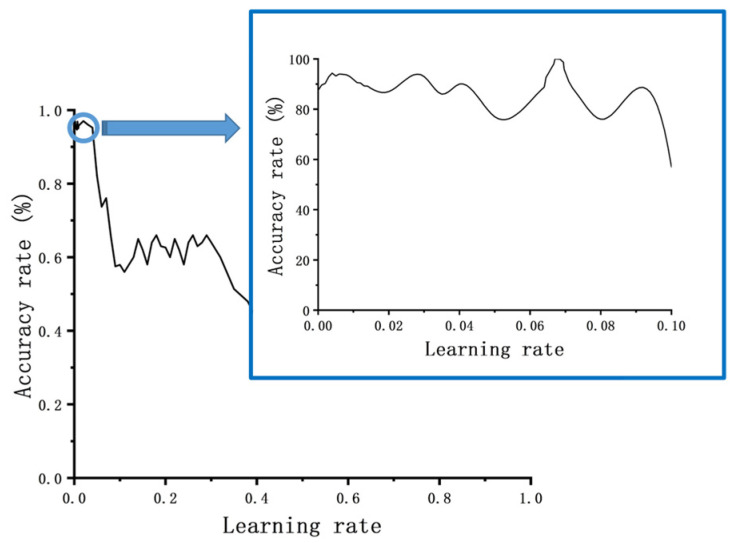
Schematic diagram of the relationship between accuracy and learning rate.

Through experimental analysis, it was observed that the best learning rate is accompanied by a range of values that yield better results, as shown in Figure 4. However, traditional methods such as using empirical values or a constant step size approach can lead to convergence at local optimal solutions (Figure 4), missing the global optimal learning rate (Figure 4). Furthermore, traditional training methods often require reducing the step size, resulting in longer training cycles and lower efficiency.

To address these challenges, this paper proposes a variable-step learning rate optimization strategy based on analyzing the relationship between the learning rate and verification set accuracy. The core idea of this strategy is to use interval subdivision, where the accuracy coefficient of each interval is represented by the training results within that interval. Before reaching the target accuracy, the local optimal solutions on both sides of the current optimization round are selected as the next target interval. This process continues until the desired accuracy is achieved, determining the global best learning rate corresponding to that target accuracy.

By adopting this variable-step learning rate optimization strategy, the paper aims to maximize accuracy while avoiding the limitations of traditional methods. The strategy leverages the concept of interval subdivision and makes use of the training results within each interval to iteratively refine and determine the optimal learning rate for achieving the desired accuracy.

To efficiently and accurately obtain the optimal learning rate parameters, this study uses the accuracy of the verification set as a metric for optimizing the variable-step learning rate. By focusing on maximizing the accuracy on the verification set, the model training process becomes more effective in identifying the best learning rate parameters. This optimization approach ensures that the model is trained with the most suitable learning rate values, leading to improved efficiency and better performance.

### 2.7. Variable-Step Learning Rate Selection Rule

The overall interval of the learning rate (lr) is (0, 1]. Since the experience interval of the common learning rate is [10^−5^, 0.1], this paper focuses on the selection of learning rate (lr) in the interval [10^−5^, 1]: within the range [10^−5^, 0.1], the learning rate adopts the selection method of variable amplitude and small step length; in the remaining interval (0.1, 1], the learning rate selection method with a long and constant amplitude is adopted.

Large interval’s fast positioning:

The learning rate is selected with variable step size in the interval [10^−5^, 0.1], and the learning rate selection formula is as follows:

(2) $${lr}_{1}=\frac{k_{2}}{10^{n}}$$

$k_{2}$ is the maximum value of the overall interval of learning rate 1. The larger parameter n is, the smaller the step size is. Parameter n is a positive integer and is selected in the range of [1, 5].

In the interval (0.1, 1], the learning rate is selected by choosing the equal step size $s_{1}$, and the formula is as follows:

(3) $$s_{1}=\frac{k_{2}-k_{1}}{p}$$

(4) $${lr}_{2}=m\times s_{1}$$

$k_{1}$ is the maximum value of the learning rate experienced at interval 0.1; $k_{2}$ is the maximum value of the learning rate of overall interval 1; parameter p is a positive integer 9; parameter m is a positive integer, and is selected in the range of [2, 10] accordingly.

Accurate optimization between cells:

The accurate optimization between cells is based on the fast positioning of large intervals. The network model is trained according to each learning rate in the large-interval fast positioning stage as a hyper parameter. Based on the learning rate ${lr}_{b}$ corresponding to the maximum accuracy in the training results, select the left-side learning rate of the benchmark ${lr}_{l}$, and right-side learning rate ${lr}_{r}$, as inter-cell lvalue and rvalue, respectively. In this interval, precise optimization is carried out.

The exact optimization between cells takes $s_{2}$ as the step size, and the learning rate is selected by the following formula:

When ${lr}_{b}-{lr}_{l}\leq {10k}_{m}$

(5) $$s_{2}=k_{m}$$

(6) $${{lr}_{1}}^{*}={lr}_{l}+s_{2}$$

$k_{m}$ is the learning rate precision, and ${lr}_{1}^{*}$ is the updated learning rate value.

When ${lr}_{b}-{lr}_{l}\geq {10k}_{m}$

(7) $$s_{2}=\frac{{lr}_{b}-{lr}_{l}}{p}$$

(8) $${lr}_{1}^{*}={lr}_{l}+s_{2}$$

The parameter p is a positive integer 9, where ${lr}_{1}^{*}$ is the updated learning rate.

Similarly, when ${lr}_{r}-{lr}_{b}\leq {10k}_{m}$

(9) $$s_{2}=k_{m}$$

(10) $${{lr}_{2}}^{*}={lr}_{b}+s_{2}$$

$k_{m}$ is the learning rate precision, and ${lr}_{2}^{*}$ is the updated learning rate value.

When ${lr}_{r}-{lr}_{b}\geq {10k}_{m}$

(11) $$s_{2}=\frac{{lr}_{r}-{lr}_{b}}{p}$$

(12) $${lr}_{2}^{*}={lr}_{b}+s_{2}$$

The parameter p is a positive integer 9, where ${lr}_{2}^{*}$ is the updated learning rate.

### 2.8. Variable-Step Size Learning Rate Optimization Process

**Figure 5:**
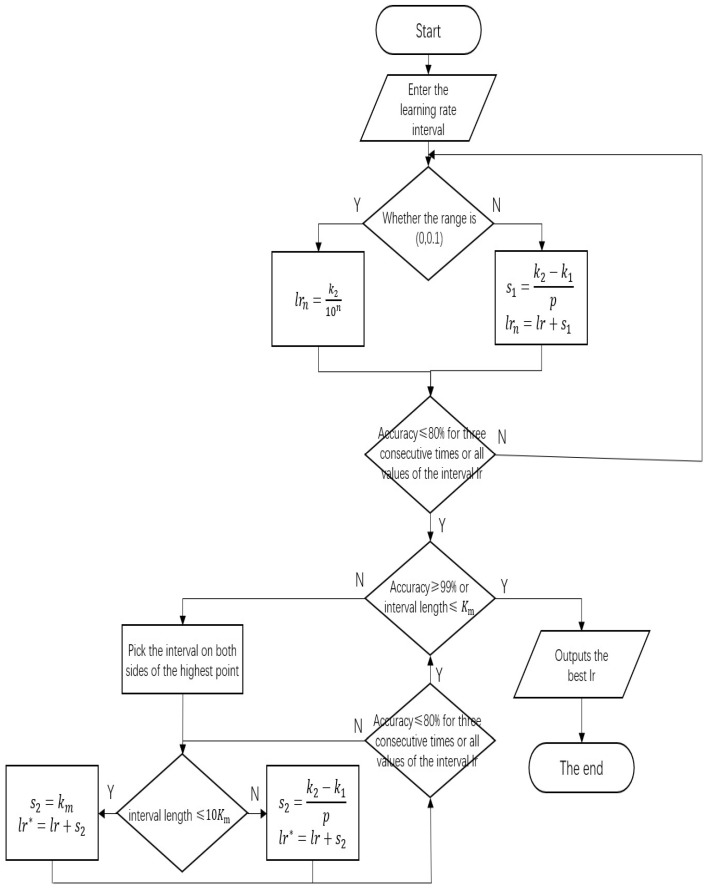
Learning rate optimization flowchart.

If the accuracy rate is lower than 80% for three consecutive times or the interval learning rate takes full value, the round optimization is interrupted and the next round optimization starts. When the interval length is less than or equal to $k_{m}$ or the accuracy of the verification set is higher than 99%, the optimization ends, and the optimal learning rate is the output. When the interval length is greater than $k_{m}$ or the accuracy of the verification set is less than 99%, the optimization continues, and the steps of inter-cell precise optimization are repeated. Figure 5 shows the global optimization process.

## 3. Experiments and Analysis

Taking the learning rate precision $k_{m}$ = 0.005 as an example, the variable-step learning rate optimization is carried out on the ResNet18 model. The experimental results are as follows:

Large interval’s fast positioning: the initial interval of learning rate is [10^−5^, 1], and the learning rate is selected according to the learning rate selection rule table. The experimental data is shown in Table 2a. As shown, when the learning rate was 0.1, 0.2, and 0.3, the accuracy of the verification set was less than 80% for three consecutive times, and the round of optimization was interrupted. The learning rate around 0.01, when the accuracy was the highest, was selected as the interval for the next round of optimization.

Accurate optimization between cells: The rule table was selected according to the learning rate, and the learning rate of 0.005 was selected for the experiment in the interval [0.001, 0.01], and each learning rate was selected for the experiment with the step size of 0.01 in the interval [0.01, 0.1). The experimental data are shown in Table 2b. When the learning rate was 0.04, 0.05, 0.06, the accuracy of the verification set was less than 80% for three consecutive times, and the round of optimization was interrupted. The interval around 0.01 of the learning rate, when the accuracy was the highest, was selected as the interval for the next round of optimization. In the next round of optimization, each learning rate was selected in the interval of [0.006, 0.02] with a step size of 0.005 for the experiment, and the experimental data is shown in Table 2c. As shown, when the learning rate obtained all values in this interval with a step size of 0.005, the optimization was stopped.

Experimental results demonstrate that the optimal learning rate for the ResNet18 model was determined to be 0.01, achieving an accuracy of 96.33%. Using a similar experimental approach, the ShuffleNet model achieved an accuracy of 96.74% with an optimal learning rate of 0.015. The corresponding experimental data are provided in Table 3.

Under the same experimental setup, the EfficientNet model reached an accuracy of 97.15% with an optimal learning rate of 0.001. Its detailed experimental data are recorded in Table 4.

The MobileNetV3 model achieved a final accuracy of 86.73% with an optimal learning rate of 0.001. Its performance metrics are summarized in Table 5.

The YOLOv8 model attained an accuracy of 88.49% under an optimal learning rate of 0.006. The complete experimental data can be found in Table 6.

To validate the effectiveness of the variable-step learning rate optimization strategy under different target accuracy requirements, experiments were conducted using the ResNet18 and ShuffleNet models. The target accuracy values for the learning rate were set to 0.001, 0.002, 0.003, and 0.004. The number of optimization iterations required using a fixed-step learning rate under the same target accuracy conditions was compared with the results obtained using the variable-step learning rate optimization strategy. The results of the comparison are presented in Table 7 and Table 8.

On this basis, three additional deep learning models—EfficientNet, MobileNetV3, and YOLOv8—were further employed for comparative verification. Under the same target accuracy conditions of 0.001, 0.002, 0.003, and 0.004, each model was optimized using both fixed-step and variable-step learning rate strategies, and the required number of iterations was recorded. The comparative results for the EfficientNet model are shown in Table 9; the data for the MobileNetV3 model are recorded in Table 10; and the comparison results for the YOLOv8 model are detailed in Table 11.

Under identical conditions regarding the number of optimizations, optimization range, and initial values, the verification accuracy of the ResNet18, ShuffleNet, EfficientNet, MobileNetV3, and YOLOv8 models trained with five different optimization methods was compared. Based on the results, it is demonstrated that the variable-step learning rate optimization strategy can rapidly achieve higher verification accuracy. The experimental conditions and results are provided in Table 12.

320 test set images were used to verify the training effect of the ResNet18 model. At the same time, the classification probability of the softmax layer was extracted and analyzed statistically, and the maximum probability of three classification labels was designed with 0.95 as the threshold. On the basis of the experimental data, the confusion matrix of four kinds of interfering fish such as bass, snapper, and cod was established, and the accuracy and recall rate of each classification were calculated. At the same time, $F_{1}$ was measured to evaluate the model comprehensively. Precision, recall, and $F_{1}$ are calculated as follows:

(13) $$P=\frac{TP}{TP+FP}$$

(14) $$R=\frac{TP}{TP+FN}$$

(15) $$F_{1}=\frac{2PR}{P+R}$$

True positives (TP) represent the number of fish correctly predicted as belonging to a certain class. False positives (FP) represent the number of fish from other classes incorrectly predicted as that class. The F1 score combines precision and recall into a single metric to measure the overall effectiveness of the model [17].

The confusion matrices and relevant parameters for the four categories of training results from the five models are shown in the table below.

## 4. Discussion

As observed from Table 7 to Table 11, under higher target accuracy requirements, the advantage of using the variable-step learning rate optimization strategy in reducing the number of optimization iterations becomes more pronounced. The reduction in global optimization iterations can reach up to 97.1%, while the reduction in empirical intervals can achieve up to 73%. This demonstrates the strategy’s ability to accurately and effectively determine the optimal learning rate.

Table 12 shows that the verification accuracy achieved using the variable-step optimization strategy is significantly higher than that achieved through constant-step optimization. The difference in accuracy between the two methods becomes more pronounced with fewer optimization iterations, demonstrating an improvement range exceeding 34.21%. Therefore, the variable-step optimization strategy enables rapid improvement in verification accuracy while minimizing the number of optimization iterations.

As shown in Table 13, the ResNet18, ShuffleNet, EfficientNet, MobileNetV3, and YOLOv8 models trained with the variable-step optimization strategy achieved average accuracy rates of 93.13%, 93.44%, 96.13%, 95.21%, 92.16%, respectively, on a test set of 320 images. These experimental results demonstrate that the models can accurately identify target fish species and distinguish them from other interfering species, meeting the requirements for practical applications.

**Figure 6:**
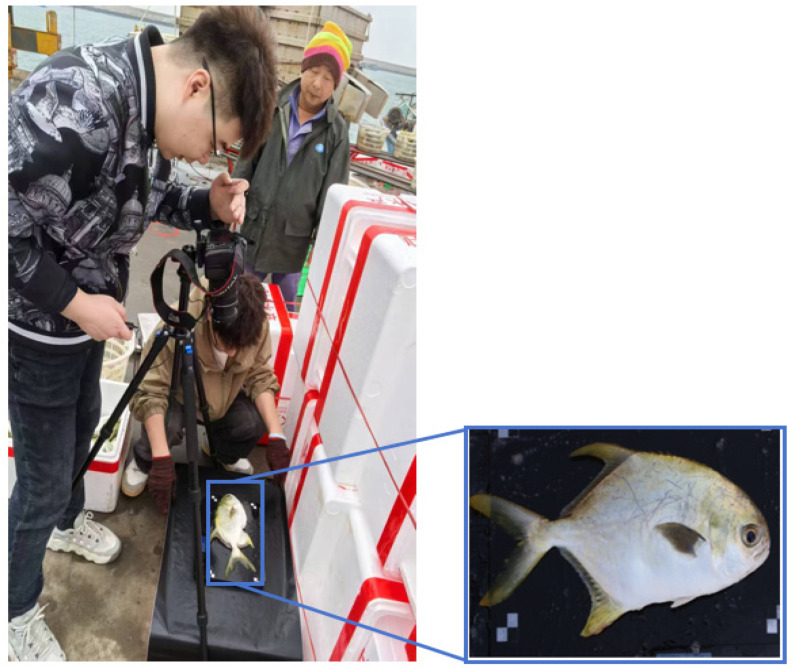
Field-collected images.

To validate the feasibility of this study, 100 images of golden pompano were collected at a fishery wharf in Fish Wharf for enhanced training of the model. The on-site data collection scenario is shown in Figure 6.

Based on previous conclusions, the corresponding interval near the learning rate of 0.01 to 0.02 was selected for additional training experiments. The model training process and performance were recorded and analyzed, and the accuracy rates at the corresponding learning rates, as shown in the Table 14, Table 15, Table 16, Table 17 and Table 18, were obtained.

The colored portions indicate the accuracy rates under optimal learning rates.

According to the data in the table, the precision, recall, and F1 score corresponding to the highest accuracy rate were selected, as shown in Table 19.

Highly valuable data and conclusions have been provided by the field experiment conducted in Hainan. Additional training experiments were performed following the aforementioned method using a portable and relatively low-configuration PC, with data collected from golden pompano samples acquired on-site in Hainan. It is shown by the experimental results that optimal accuracy was achieved by the five lightweight models in approximately 30 min, with time and application costs significantly reduced compared to traditional large models. This advantage is particularly evident in offshore fishing scenarios: multiple constraints are imposed by the maritime operating environment, making it difficult for high-performance devices capable of supporting large-scale network computations to be deployed. In contrast, notable practicality and adaptability are demonstrated by the approach adopted in this experiment.

Furthermore, through this golden pompano training experiment, the exceptional performance of the variable-step size learning rate optimization strategy in practical applications was demonstrated. Not only was the theoretical soundness of this strategy confirmed, but strong feasibility and generalization capability were also exhibited, effectively supporting model training in real-world environments.

This study has several limitations. First, although lightweight network architectures were employed, the practical deployment performance of the models on low-configuration hardware devices has not been fully validated. Key metrics such as real-time inference speed, memory usage, and power consumption lack systematic evaluation, which somewhat undermines the reliability of the models in real-world fishery scenarios. Second, several parameters in the proposed variable-step size learning rate optimization strategy—such as interval division thresholds and precision coefficients—still rely on empirical settings rather than theoretical derivation or adaptive mechanisms. Therefore, the generalizability of this strategy across different network architectures and tasks requires further verification. Additionally, the limited scale and diversity of the training data may increase the risk of overfitting. This is reflected in the relatively low accuracy of some models on the validation set (e.g., MobileNetV3 at 86.73%), indicating that the models may be overly dependent on specific features of the training set, while their generalization capability remains to be improved.

## 5. Conclusions

To address fishing challenges in harsh environments, this study tackles issues such as prolonged training cycles, complex training processes, and dynamic classification requirements. By employing lightweight network models including ResNet18, ShuffleNet, EfficientNet, MobileNetV3, and YOLOv8 for transfer learning, it resolves the complexity of mixed-species fishing in the Bohai and Yellow Sea regions. Furthermore, an intelligent fish classification method based on a variable-step learning rate optimization strategy is proposed, utilizing a dataset of 2289 images featuring snapper, cod, and sea bass species.

Experimental results show that when the target accuracy requirement for the learning rate is set to the optimal value, the verification accuracy of ResNet18, ShuffleNet, EfficientNet, MobileNetV3, and YOLOv8 reaches 96.33%, 96.74%, 97.50%, 86.73%, and 88.49%, respectively. On a test set of 320 images containing other interfering fish species, the average accuracy reaches 93.13%, 93.44%, 96.13%, 95.21%, and 92.16%, respectively. By adopting a variable-step size learning rate optimization strategy, the number of optimization iterations is reduced by more than 97.1% compared to global theoretical optimization and by more than 73% compared to empirical interval optimization.

The experimental results demonstrate the effectiveness of the proposed intelligent fish sorting method based on the variable-step learning rate optimization strategy. This method provides a theoretical foundation and technical support for automatic online fish sorting in fisheries. Moreover, the proposed strategy is not dependent on the specific network model’s structure or characteristics. It can effectively reduce parameter optimization efforts and improve model accuracy across different network models. This has significant implications for enhancing training efficiency and shortening the overall training period.

## Figures and Tables

**Table 1:** Dataset composition.

Number of Datasets (Sheets)	Perch	Snapper	Cod	Other Multi-Species Fish
Training set	734	292	452	none
Verification set	244	97	150	none
Test set	80	80	80	80

**Table 2:** Experimental data of learning rate selection for ResNet18.

a. The first round of experimental data																					
**Learning rate**	**10^−5^**	**10^−4^**	**10^−3^**	**0.01**	**0.1**	**0.2**	**0.3**	**0.4**		**0.5**		**0.6**		**0.7**		**0.8**		**0.9**		**1**	
Verification set accuracy	47.45%	86.35%	93.48%	96.33%	69.86%	56.21%	59.88%														
													
b. Experimental data of the second round																					
**Learning rate**	**0.001**		**0.006**	**0.01**	**0.02**	**0.03**	**0.04**	**0.05**		**0.06**		**0.07**		**0.08**		**0.09**		**0.1**			
Verification set accuracy	93.48%		96.13%	96.33%	95.72%	91.85%	79.02%	77.60%		71.08%											
							
c. Data from the third round of experiments																					
**Learning rate**			**0.006**			**0.01**		**0.015**				**0.02**									
Verification set accuracy			96.13%			96.33%		95.72%				95.72%									

The color annotations indicate the next-step learning rate adjustment intervals and the optimal learning rate intervals. All subsequent tables follow this convention.

**Table 3:** Experimental data of learning rate selection for ShuffleNet.

a. The first round of experimental data																
**Learning rate**	**10^−5^**	**10** **^−^** **^4^**	**10** **^−^** **^3^**	**0.01**	**0.1**	**0.2**	**0.3**	**0.4**	**0.5**	**0.6**	**0.7**	**0.8**	**0.9**		**1**	
Verification set accuracy	48.47%	80.45%	96.26%	96.54%	85.34%	83.10%	79.84%	80.04%	81.06%	77.39%	76.58%	75.15%				
			
b. Experimental data of the second round																
**Learning rate**		**0.001**	**0.006**	**0.01**	**0.02**	**0.03**	**0.04**	**0.05**	**0.06**	**0.07**	**0.08**	**0.09**	**0.1**			
Verification set accuracy		92.26%	95.72%	96.54%	95.32%	95.11%	90.43%	83.71%	78.62%	83.91%	87.37%	86.56%	85.34%			
c. Data from the third round of experiments																
**Learning rate**		**0.006**			**0.01**		**0.015**			**0.02**						
Verification set accuracy		95.72%			96.54%		96.74%			95.32%						

**Table 4:** Experimental data of learning rate selection for EfficientNet.

a. The first round of experimental data																					
**Learning rate**	**10^−5^**	**10** **^−^** **^4^**	**10** **^−^** **^3^**	**0.01**	**0.1**	**0.2**	**0.3**	**0.4**		**0.5**		**0.6**		**0.7**		**0.8**		**0.9**		**1**	
Verification set accuracy	76.51%	98.00%	97.50%	92.00%	71.75%	69.00%	49.50%														
													
b. Experimental data of the second round																					
**Learning rate**	**0.001**	**0.006**	**0.01**	**0.02**	**0.03**	**0.04**	**0.05**	**0.06**		**0.07**		**0.08**		**0.09**		**0.1**					
Verification set accuracy	97.50%	95.25%	92.00%	87.25%	88.75%	80.25%	78.75%	74.00%													
											
c. Data from the third round of experiments																					
**Learning rate**				**0.0** **005**			**0.0** **01**			**0.0** **015**				**0.0** **02**							
Verification set accuracy				96.13%			97.50%			95.72%				93.72%							

**Table 5:** Experimental data of learning rate selection for MobileNetV3.

a. The first round of experimental data																		
**Learning rate**	**10^−5^**	**10** **^−^** **^4^**	**10** **^−^** **^3^**	**0.01**	**0.1**	**0.2**	**0.3**	**0.4**	**0.5**	**0.6**	**0.7**		**0.8**		**0.9**		**1**	
Verification set accuracy	50.40%	77.00%	79.65%	84.07%	42.48%	46.02%	46.90%	47.79%	37.17%	35.13%								
							
b. Experimental data of the second round																		
**Learning rate**	**0.001**		**0.006**	**0.01**	**0.02**	**0.03**	**0.04**	**0.05**	**0.06**	**0.07**	**0.08**		**0.09**		**0.1**			
Verification set accuracy	86.73%		77.88%	84.07%	55.75%	53.10%	52.22%	58.41%	53.98%	47.79%	54.39%		46.02%					
			
c. Data from the third round of experiments																		
**Learning rate**				**0.0005**		**0.001**			**0.0015**				**0.02**					
Verification set accuracy				84.96%		86.73%			86.61%				55.75%					

**Table 6:** Experimental data of learning rate selection for YOLOv8.

a. The first round of experimental data																						
**Learning rate**	**10^−5^**	**10** **^−^** **^4^**	**10** **^−^** **^3^**	**0.01**	**0.1**	**0.2**	**0.3**		**0.4**		**0.5**		**0.6**		**0.7**		**0.8**		**0.9**		**1**	
Verification set accuracy	51.33%	82.30%	87.50%	47.79%	22.12%	23.10%																
															
b. Experimental data of the second round																						
**Learning rate**	**0.0006**	**0.001**	**0.01**	**0.02**	**0.03**	**0.04**	**0.05**		**0.06**				**0.07**		**0.08**		**0.09**				**0.1**	
Verification set accuracy	88.49%	87.31%	84.07%	44.24%	44.25%	37.16%	20.35%		19.47%				15.93%									
							
c. Data from the third round of experiments																						
**Learning rate**			**0.0004**			**0.006**			**0.001**						**0.0015**							
Verification set accuracy			87.23%			88.49%			87.31%						84.95%							

**Table 7:** Comparison of learning rate optimization times for ResNet18.

Learning Rate Optimization Interval	Target Precision	Variable-Step Size Optimization Theoretical Optimization Times/Times	Variable-Step Size Optimization Actual Optimization Times/Times	Theoretical Optimization Times/Times	The Reduction in Actual Optimization Time
(0, 1] Global optimization	0.001	39	29	1000	97.1%
	0.002	30	20	500	96.0%
	0.003	27	17	333	94.9%
	0.004	26	16	250	93.6%
	0.005	24	14	200	93.0%
(0, 0.1] Optimization of experience interval	0.001	30	27	100	73.0%
	0.002	21	18	50	64.0%
	0.003	18	15	33	54.5%
	0.004	17	14	25	44.0%
	0.005	15	12	20	40.0%

**Table 8:** Comparison of learning rate optimization times for ShuffleNet.

Learning Rate Optimization Interval	Target Precision	Variable-Step Size Optimization Theoretical Optimization Times/Times	Variable-Step Size Optimization Actual Optimization Times/Times	Theoretical Optimization Times/Times	The Reduction in Actual Optimization Time
(0, 1] Global optimization	0.001	39	37	1000	96.3%
	0.002	30	28	500	94.4%
	0.003	27	25	333	92.5%
	0.004	26	24	250	90.4%
	0.005	24	22	200	89.0%
(0, 0.1] Optimization of experience interval	0.001	30	30	100	70.0%
	0.002	21	21	50	58.0%
	0.003	18	18	33	45.5%
	0.004	17	17	25	32.0%
	0.005	15	15	20	25.0%

**Table 9:** Comparison of learning rate optimization times for EfficientNet.

Learning	Target Precision	Variable-Step Size Optimization Theoretical Optimization Times/Times	Variable-Step Size Optimization Actual Optimization Times/Times	Theoretical Optimization Times/Times	The Reduction in Actual Optimization Time
(0, 1] Global optimization	0.001	39	35	1000	96.5%
	0.002	30	29	500	94.2%
	0.003	27	21	333	93.7%
	0.004	26	15	250	94.0%
	0.005	24	13	200	93.5%
(0, 0.1] Optimization of experience interval	0.001	30	28	100	72.0%
	0.002	21	19	50	62.0%
	0.003	18	14	33	57.6%
	0.004	17	13	25	48.0%
	0.005	15	11	20	45.0%

**Table 10:** Comparison of learning rate optimization times for MobileNetV3.

Learning Rate Optimization Interval	Target Precision	Variable-Step Size Optimization Theoretical Optimization Times/Times	Variable-Step Size Optimization Actual Optimization Times/Times	Theoretical Optimization Times/Times	The Reduction in Actual Optimization Time
(0, 1] Global optimization	0.001	39	37	1000	96.3%
	0.002	30	24	500	95.2%
	0.003	27	19	333	94.9%
	0.004	26	17	250	94.3%
	0.005	24	16	200	92.0%
(0, 0.1] Optimization of experience interval	0.001	30	29	100	71.0%
	0.002	21	20	50	60.0%
	0.003	18	16	33	51.5%
	0.004	17	13	25	48.0%
	0.005	15	12	20	40.0%

**Table 11:** Comparison of learning rate optimization times for YOLOv8.

Learning Rate Optimization Interval	Target Precision	Variable-Step Size Optimization Theoretical Optimization Times/Times	Variable-Step Size Optimization Actual Optimization Times/Times	Theoretical Optimization Times/Times	The Reduction in Actual Optimization Time
(0, 1] Global optimization	0.001	39	32	1000	96.8%
	0.002	30	24	500	95.2%
	0.003	27	20	333	93.9%
	0.004	26	17	250	93.2%
	0.005	24	15	200	92.5%
(0, 0.1] Optimization of experience interval	0.001	30	28	100	72.0%
	0.002	21	20	50	60.0%
	0.003	18	17	33	48.5%
	0.004	17	16	25	36.0%
	0.005	15	13	20	35.0%

**Table 12:** Comparison of verification sets accuracy results between two methods.

a. ResNet18			
**Optimization times/times**	**Equal-step**	**Variable-step**	**Accuracy improvement**
5	62.12%	96.33%	34.21%
10	76.37%	96.33%	19.96%
20	77.60%	96.33%	18.73%
b. ShuffleNet			
**Optimization times/times**	**Equal-step**	**Variable-step**	**Accuracy improvement**
5	83.10%	96.54%	13.44%
10	85.34%	96.54%	11.20%
20	85.34%	96.54%	11.20%
c. EfficientNet			
**Optimization times/times**	**Equal-step**	**Variable-step**	**Accuracy improvement**
5	78.23%	98.13%	19.90%
10	82.45%	98.13%	15.68%
20	85.60%	98.13%	12.53%
d. MobileNetV3			
**Optimization times/times**	**Equal-step**	**Variable-step**	**Accuracy improvement**
5	59.22%	86.88%	27.66%
10	66.24%	86.88%	20.64%
20	71.61%	86.88%	15.27%
e. YOLOv8			
**Optimization times/times**	**Equal-step**	**Variable-step**	**Accuracy improvement**
5	61.34%	88.49%	25.54%
10	70.42%	88.49%	16.46%
20	76.65%	88.49%	10.23%

**Table 13:** Confusion matrix and evaluation results.

a. Four-category confusion matrix (ResNet18)				
	**Perch**	**Snapper**	**Cod**	**Other multiple species fish**
TP	80	78	73	67
FP	6	4	3	9
FN	0	2	7	13
b. Accuracy rate and recall rate of the four categories (ResNet18)				
	**Perch**	**Snapper**	**Cod**	**Other multiple species fish**
P	0.930	0.951	0.961	0.882
R	1.000	0.975	0.913	0.838
$F_{1}$	0.964	0.963	0.936	0.859
c. Four-category confusion matrix (ShuffleNet)				
	**Perch**	**Snapper**	**Cod**	**Other multiple species fish**
TP	77	77	76	69
FP	6	0	5	10
FN	3	3	4	11
d. Accuracy rate and recall rate of the four categories (ShuffleNet)				
	**Perch**	**Snapper**	**Cod**	**Other multiple species fish**
P	0.928	1.000	0.938	0.873
R	0.963	0.963	0.950	0.863
$F_{1}$	0.945	0.981	0.944	0.868
e. Four-category confusion matrix (EfficientNet)				
	**Perch**	**Snapper**	**Cod**	**Other multiple species fish**
TP	75	80	78	75
FP	80	0	5	0
FN	0	0	2	5
f. Accuracy rate and recall rate of the four categories (EfficientNet)				
	**Perch**	**Snapper**	**Cod**	**Other multiple species fish**
P	0.9756	1.0000	0.9398	1.0000
R	1.0000	1.0000	0.9750	0.9375
$F_{1}$	0.9877	1.0000	0.9571	0.9677
g. Four-category confusion matrix (MobileNetV3)				
	**Perch**	**Snapper**	**Cod**	**Other multiple species fish**
TP	80	80	80	72
FP	4	0	4	0
FN	0	0	0	8
h. Accuracy rate and recall rate of the four categories (MobileNetV3)				
	**Perch**	**Snapper**	**Cod**	**Other multiple species fish**
P	0.952	1.000	0.952	1.000
R	1.000	1.000	1.000	0.912
$F_{1}$	0.976	1.000	0.976	0.947
i. Four-category confusion matrix (YOLOv8)				
	**Perch**	**Snapper**	**Cod**	**Other multiple species fish**
TP	80	77	79	64
FP	2	0	17	1
FN	0	3	1	16
j. Accuracy rate and recall rate of the four categories (YOLOv8)				
	**Perch**	**Snapper**	**Cod**	**Other multiple species fish**
P	0.975	1.000	0.829	0.984
R	1.000	0.962	0.987	0.819
$F_{1}$	0.988	0.981	0.897	0.883

**Table 14:** Experimental data of learning rate selection for ResNet18.

Learning Rate	0.015	0.018	0.02
verification set accuracy	92.22%	96.67%	96.18%

**Table 15:** Experimental data of learning rate selection for ShuffleNet.

Learning Rate	0.008	0.01	0.015
verification set accuracy	94.67%	98.18%	94.29%

**Table 16:** Experimental data of learning rate selection for EfficientNet.

Learning Rate	0.015	0.001	0.06
verification set accuracy	94.44%	96.67%	94.43%

**Table 17:** Experimental data of learning rate selection for MobileNetV3.

Learning Rate	0.0005	0.001	0.0015
verification set accuracy	83.19%	85.96%	84.83%

**Table 18:** Experimental data of learning rate selection for YOLOv8.

Learning Rate	0.005	0.006	0.007
verification set accuracy	86.67%	91.11%	90.12%

**Table 19:** Evaluation results.

a. Accuracy rate and recall rate of the five categories (ResNet18)					
	**Perch**	**Snapper**	**Cod**	**Other multiple species fish**	**Golden Pomfret**
P	1.000	0.833	1.000	1.000	1.000
R	1.000	1.000	0.900	0.900	1.000
$F_{1}$	1.000	0.909	0.909	0.947	1.000
b. Accuracy rate and recall rate of the five categories (ShuffleNet)					
	**Perch**	**Snapper**	**Cod**	**Other multiple species fish**	**Golden Pomfret**
P	1.000	1.000	0.909	1.000	1.000
R	1.000	1.000	1.000	0.900	1.000
$F_{1}$	1.000	1.000	0.952	0.947	1.000
c. Accuracy rate and recall rate of the five categories (EfficientNe)					
	**Perch**	**Snapper**	**Cod**	**Other multiple species fish**	**Golden Pomfret**
P	1.000	1.000	1.000	0.932	1.000
R	1.000	0.912	0.923	0.912	1.000
$F_{1}$	1.000	1.000	1.000	0.952	0.947
d. Accuracy rate and recall rate of the five categories (MobileNetV3)					
	**Perch**	**Snapper**	**Cod**	**Other multiple species fish**	**Golden Pomfret**
P	1.000	0.933	0.961	0.871	0.921
R	0.943	1.000	0.941	0.931	0.921
$F_{1}$	0.912	0.923	0.939	0.947	0.941
e. Accuracy rate and recall rate of the five categories (YOLOv8)					
	**Perch**	**Snapper**	**Cod**	**Other multiple species fish**	**Golden Pomfret**
P	0.987	0.976	0.842	0.870	0.978
R	0.950	1.000	0.863	0.837	1.000
$F_{1}$	0.968	0.976	0.852	0.854	0.989

## Data Availability

The original contributions presented in the study are included in the article, further inquiries can be directed to the corresponding authors.
